# Supplementary material for: Effect of Systemic Steroid Use for Immune-Related Adverse Events in Patients with Non-Small Cell Lung Cancer Receiving PD-1 Blockade Drugs
Source: J Clin Med. 2021 Aug 23;10(16):3744. doi: 10.3390/jcm10163744 (PMC8397225; doi:10.3390/jcm10163744)
Supplement: Supplementary file 1 [file jcm-10-03744-s001.zip › jcm-1327254-supplementary.pdf]

(A) (B)

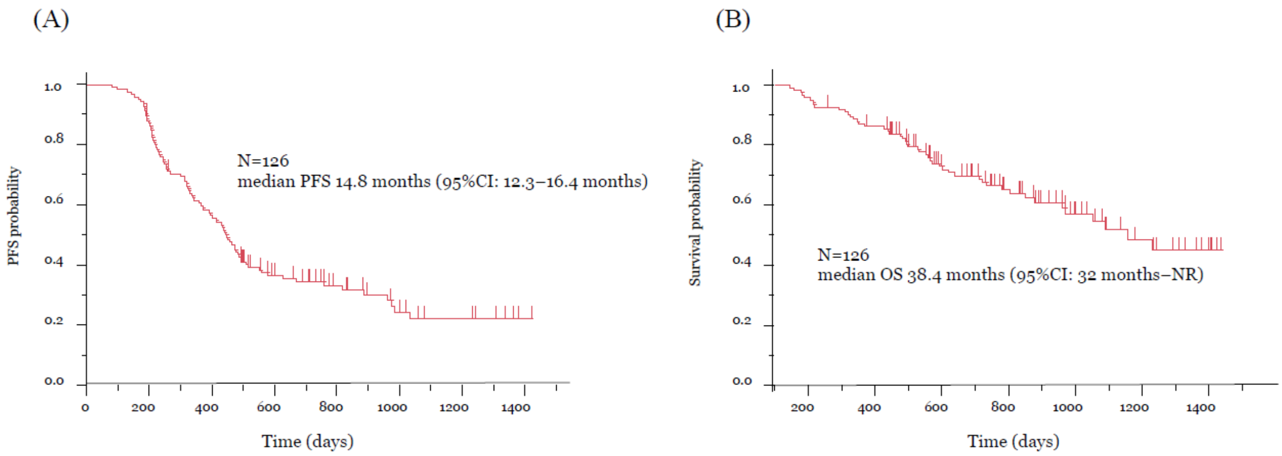

Figure S1. Kaplan–Meier survival analysis in all target patients defined as responders. The figures show Kaplan–Meier curves for PFS (S1A) and OS (S1B) in all target patients defined as responders. PFS, progression-free survival; OS, overall survival; NR, not reached.
